# Antibiotic Use and Antibiotic Susceptibility of Common Environmental Bacterial Species in the Intensive Care Unit at Mbarara Regional Referral Hospital, Uganda

**DOI:** 10.7759/cureus.106696

**Published:** 2026-04-09

**Authors:** Daniel Chans Mwandah, Martha K Namakula, Joshua Kiprotich, Stella Babirye, Namuyomba M Kamoga, Splendour Masinde, Anna B Nakiguli, Kevin Kiyimba, Nuru Mugide, Tadele M Yadesa, Jonans Tusiimire, Stephen Ttendo, Joseph Oloro, Joel Bazira, Amon G Agaba

**Affiliations:** 1 Department of Pharmacology and Therapeutics, Mbarara University of Science and Technology, Mbarara, UGA; 2 Department of Pharmacology and Toxicology, Kampala International University-Western Campus (WC), Bushenyi, UGA; 3 Department of Pharmacy, Mbarara University of Science and Technology, Mbarara, UGA; 4 Department of Clinical Pharmacy and Pharmacy Practice, Kampala International University-Western Campus (WC), Bushenyi, UGA; 5 Faculty of Clinical Medicine and Dentistry, Kampala International University-Western Campus (WC), Bushenyi, UGA; 6 Department of Anaesthesia and Critical Care, Mbarara University of Science and Technology, Mbarara, UGA; 7 Department of Microbiology and Parasitology, Mbarara University of Science and Technology, Mbarara, UGA

**Keywords:** antibiotic susceptibility, antimicrobial resistance, environmental contamination, intensive care unit, multidrug-resistant bacteria

## Abstract

Background: Environmental contamination in intensive care units (ICUs) is a recognized concern due to its potential role as a reservoir for pathogenic and antimicrobial-resistant bacteria. Understanding the extent and patterns of contamination is essential for strengthening infection prevention and control strategies, particularly in resource-limited settings.

Methods: A cross-sectional environmental surveillance study was conducted in the ICU of Mbarara Regional Referral Hospital. A total of 34 high-touch surfaces were sampled using sterile pre-moistened swabs over a standardized area. Bacterial identification was based on colony morphology, hemolytic patterns, Gram staining, and microscopic characteristics. Antimicrobial susceptibility testing was performed using the disc diffusion method, with zones of inhibition measured in millimeters and interpreted as sensitive, intermediate, or resistant according to standard guidelines.

Results: Of the 34 environmental samples collected, 32 (94.1%) yielded bacterial growth, resulting in a total of 40 isolates. Gram-positive bacteria accounted for 21 isolates (52.5%), while Gram-negative bacteria accounted for 19 isolates (47.5%). Common isolates included *Staphylococcus aureus*, *Klebsiella* species, and *Escherichia coli*. Antimicrobial susceptibility testing revealed varying resistance patterns across commonly used antibiotics, with notable resistance observed among several isolates. Descriptive patient data indicated frequent use of broad-spectrum antibiotics within the ICU.

Conclusions: This study demonstrates a high level of bacterial contamination on ICU surfaces, with diverse organisms exhibiting varying antimicrobial resistance patterns. While no direct relationship between environmental contamination and patient infections was established, the findings highlight the importance of maintaining stringent infection prevention and control practices. Further studies incorporating advanced microbiological techniques are recommended to better understand transmission dynamics in ICU settings. The findings highlight the urgent need for strengthened antimicrobial stewardship programs and routine culture and sensitivity testing in resource-limited ICU settings.

## Introduction

Antimicrobial resistance (AMR) is a major global public health threat, responsible for at least 1.27 million deaths worldwide and associated with nearly five million deaths in 2019 [1]. Projections from a review commissioned by the United Kingdom government estimated that AMR could result in up to 10 million deaths annually by 2050 [1]. The misuse and overuse of antimicrobials in humans, animals, and agriculture are the primary drivers of the emergence and spread of drug-resistant pathogens [2]. Despite ongoing global efforts to address AMR, data on intensive care unit (ICU)-acquired infections and AMR patterns in low-resource settings, including Uganda, remain limited.

There is high use of antibiotics in the ICU and high mortality rates among ICU patients. However, in our setting, this is not well studied. The most common ICU-acquired infections include pneumonia, particularly ventilator-associated pneumonia (VAP), surgical site infections (SSIs), catheter-related bloodstream infections (CRBSIs), and catheter-associated urinary tract infections (CAUTIs) [3,4]. Rates of ICU-associated hospital-acquired infections (HAIs) are generally higher in low- and middle-income countries compared to high-income countries, with a greater burden of multidrug-resistant organisms (MDROs), especially among Gram-negative bacteria [5,6]. ICU patients are frequently treated with broad-spectrum antibiotics, which exert selective pressure and promote the development and persistence of AMR among commonly encountered pathogens [7].

HAIs are a leading cause of morbidity and mortality in ICUs and are commonly caused by organisms such as *Enterococcus* spp., *Klebsiella* spp., *Acinetobacter* spp., *Pseudomonas aeruginosa*, and members of the Enterobacteriaceae family [8]. These pathogens can persist on environmental surfaces and medical equipment and may be transmitted through direct contact or via healthcare workers [9].

Environmental surfaces within ICUs serve as important reservoirs for pathogenic bacteria and play a significant role in the transmission of healthcare-associated infections. This study was therefore conducted to assess bacterial contamination and AMR patterns on environmental surfaces in the ICU of Mbarara Regional Referral Hospital. Additionally, descriptive patient data were collected to provide contextual insight into antibiotic use within the unit. The findings are intended to inform infection prevention and control practices and contribute to the growing body of evidence on environmental surveillance in critical care settings.

## Materials and methods

Study design, study area, and setting

A cross-sectional environmental surveillance study was conducted aimed at assessing bacterial contamination and AMR patterns on ICU surfaces of Mbarara Regional Referral Hospital (MRRH), Uganda. In addition, retrospective data on antibiotic use, patient outcomes, medical history, and sociodemographic characteristics were extracted from the medication charts of patients admitted to the ICU between January 2022 and June 2023. Patient-related data were included for descriptive purposes only and were not linked analytically to environmental isolates.

Study population

The minimum sample size of 87 patients was determined using a single population proportion formula, assuming a 95% confidence level, a two-sided margin of error of 5%, and an estimated AMR prevalence of 6% based on a previous study [10]. All patients admitted to the ICU during the study period who received at least one antibiotic were considered eligible. Patients with incomplete or illegible medical records were excluded. Clinical, medication, and treatment outcome data were collected using a pretested data collection form. Ultimately, all eligible patients encountered during the study period were included, resulting in a final sample size of 109. This larger sample enhanced the precision and robustness of the study findings.

Specimen collection

Frequently touched ICU surfaces were identified in consultation with a microbiologist and ICU nursing staff. Specimen collection was conducted on different days over a three-week period. A standardized surface area of 25 cm² was swabbed using sterile pre-moistened swabs to enhance bacterial recovery from dry surfaces. Each site was sampled once using a single swab. Swabbing was performed in both horizontal and vertical directions to ensure adequate surface coverage. Reference strains from the ATCC class (*Escherichia coli* ATCC 25922 and *Staphylococcus aureus* ATCC 25923) were used as positive controls to ensure quality assurance.

Following collection, swabs were immediately placed into sterile Bijou bottles containing brain heart infusion broth, securely sealed, labeled, and transported to the microbiology laboratory within one hour at room temperature. A total of 34 environmental samples were collected one hour after routine cleaning and disinfection. Between two and six samples were obtained per site from pre-defined high-touch surfaces, including worktops, beds, door knobs, trays, drug trolleys, sinks, taps, ventilators, and oxygen ports.

The sample size was informed by previous studies conducted in similar settings, taking into account ICU size, feasibility, and available resources. Trained and licensed laboratory technologists were used to collect the samples; they observed aseptic technique and uniform pressure during collection. Negative field controls were used to detect any environmental contamination. Samples were collected within two hours, and each batch was tested for sterility. Unused sterile swabs were sent with samples to detect any contamination during transportation. There was no cross-contamination between samples.

Bacterial isolates

Specimens were cultured using conventional culture media in accordance with the standard operating procedures of the microbiology laboratory at Mbarara University of Science and Technology. Samples were inoculated onto MacConkey agar and blood agar plates using sterile inoculation loops and incubated aerobically at 37°C for 24 hours.

Following incubation, bacterial growth was assessed based on colony morphology and density. Colonies were examined macroscopically and microscopically. Bacterial identification was performed using standard microbiological techniques, including Gram staining and biochemical testing.

Gram-negative bacteria were identified based on lactose fermentation, hydrogen sulfide production, catalase, oxidase, indole, citrate utilization, urease activity, and gas production tests. Gram-positive bacteria were identified using hemolytic patterns on blood agar, catalase testing, and coagulase testing.

Bacterial growth from environmental samples was interpreted qualitatively and semi-quantitatively based on the presence and relative abundance of colonies, rather than applying clinical diagnostic thresholds. This approach is consistent with standard environmental microbiological surveillance practices.

Quality assurance of culture media was performed by incubating 3% to 5% of each prepared batch at 35°C to 37°C for 24 hours. Media demonstrating any growth were discarded prior to use to ensure sterility and reliability of results.

Standardization and preparation of the inoculum

Bacterial inocula were standardized using a 0.5-McFarland turbidity standard prepared by mixing 0.50 mL of 1.175% barium chloride dihydrate with 99.50 mL of 1% sulfuric acid. The standard was aliquoted into test tubes, and turbidity was verified spectrophotometrically at 625 nm with an absorbance of 0.09.

Four to five morphologically similar colonies from overnight cultures were suspended in 5 mL of 0.85% normal saline and mixed using a vortex mixer. The turbidity was adjusted to match the 0.5-McFarland standard, corresponding to approximately 1.5 × 10⁸ CFU/mL. A sterile cotton swab was dipped into the suspension and used to evenly inoculate the surface of Mueller-Hinton agar plates.

Antibiotic susceptibility testing

Antimicrobial susceptibility testing was performed using the Kirby-Bauer disc diffusion method on Mueller-Hinton agar in accordance with standard microbiological procedures. The bacterial inoculum was prepared and standardized prior to inoculation to ensure uniformity.

The antibiotics tested included ceftriaxone (30 µg), gentamicin (10 µg), ciprofloxacin (5 µg), ampicillin (10 µg), imipenem (10 µg), ceftazidime (30 µg), oxacillin (1 µg), and azithromycin (30 µg). Following inoculation, plates were allowed to dry for three to five minutes before antibiotic discs were aseptically applied. The plates were then incubated aerobically at 37°C for 18 to 24 hours.

**Figure 1:**
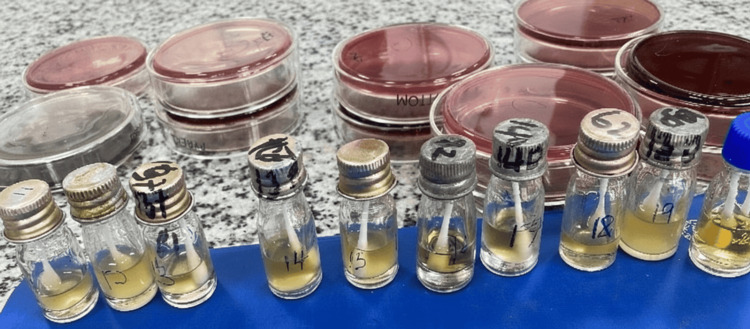
Swab sticks placed into Bijou bottles immediately after specimen collection

**Figure 2:**
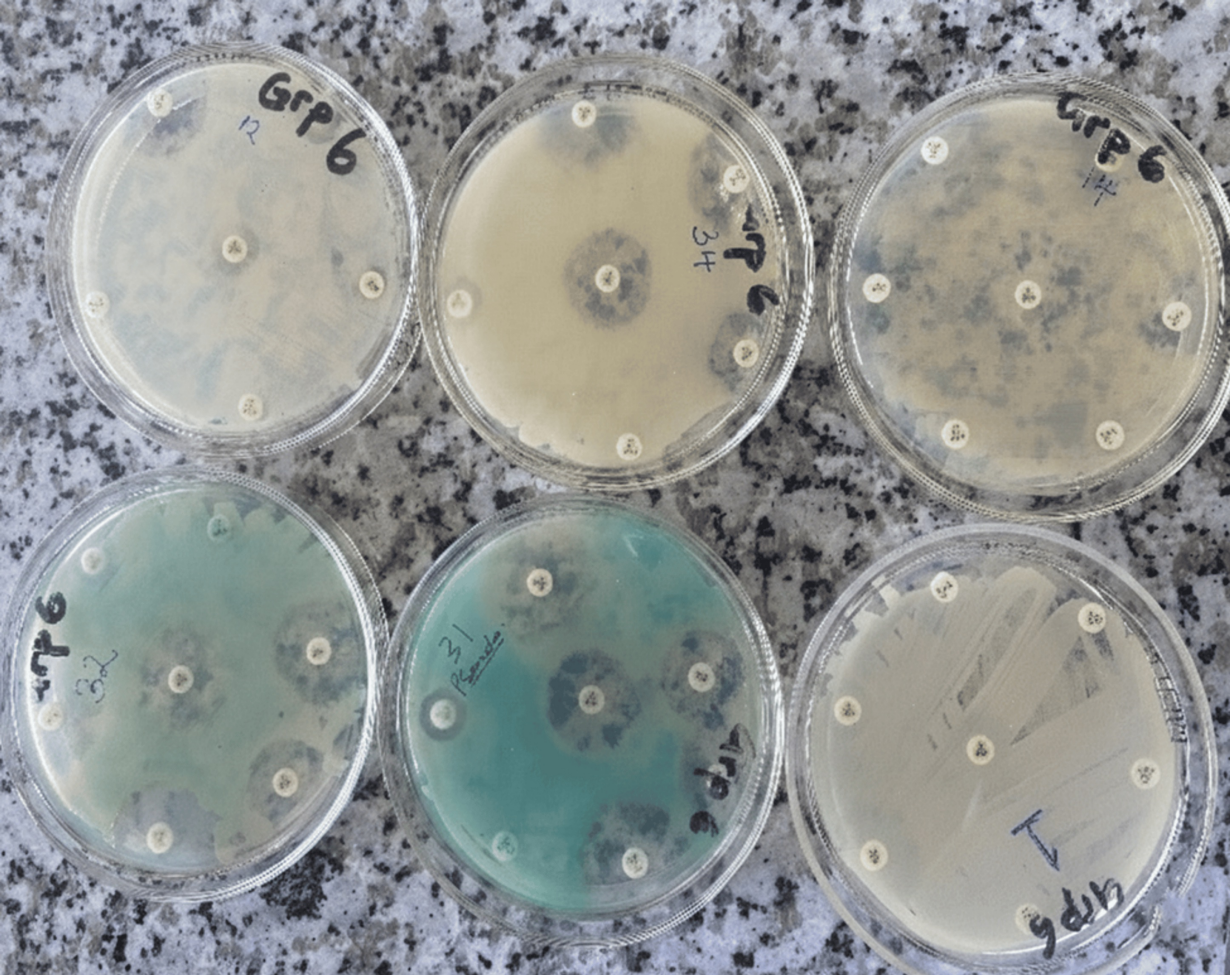
Zones of inhibition demonstrating varying bacterial susceptibility to commonly used antibiotics

**Figure 3:**
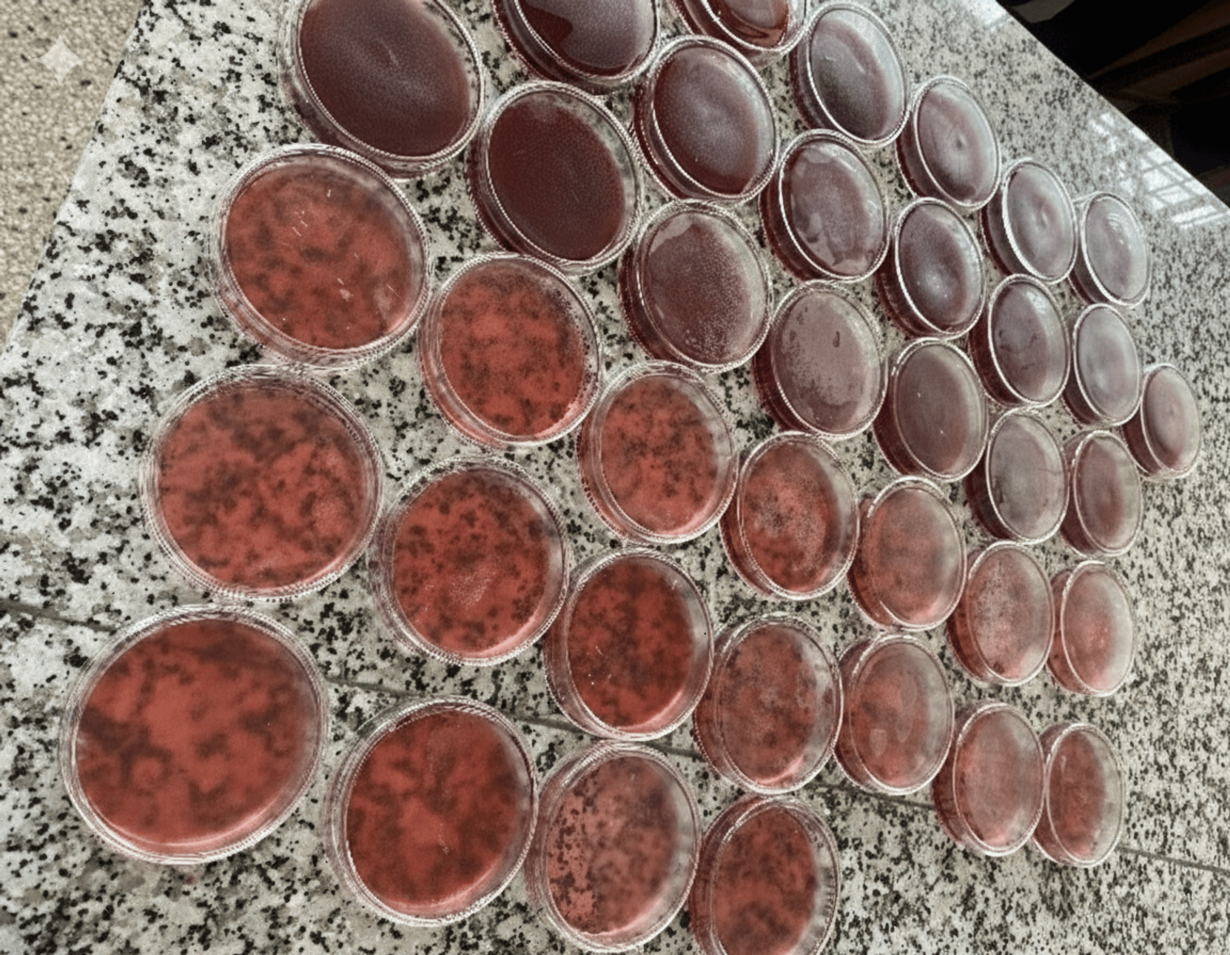
Prepared Mueller-Hinton agar and blood agar plates prior to inoculation

**Figure 4:**
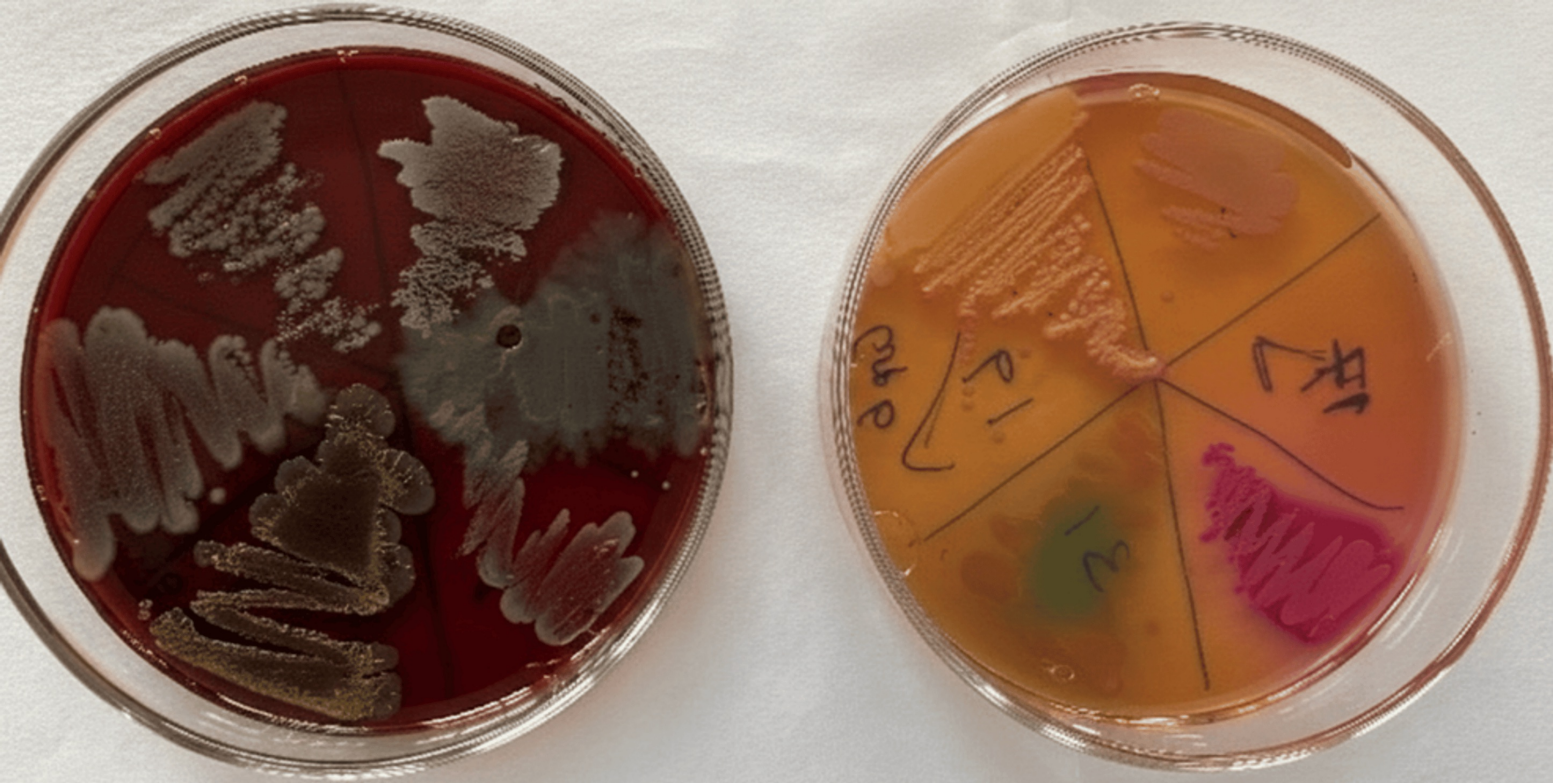
Growth of bacterial isolates on Mueller-Hinton agar and blood agar following incubation at 37°C for 24 hours

Zones of inhibition were measured in millimeters using a calibrated ruler and interpreted as susceptible, intermediate, or resistant in accordance with the Clinical and Laboratory Standards Institute guidelines [11] and European Committee on Antimicrobial Susceptibility (Figures 1-4) [12].

The antibiotic panel was selected based on a preliminary assessment of the most commonly prescribed antibiotics in the ICU setting, ensuring clinical relevance to local treatment practices. Antibiotics such as vancomycin were not included in the initial testing panel, as their use is typically reserved for specific Gram-positive organisms following identification rather than routine empirical screening.

Ethical approval

Ethical approval for this study was obtained from the Institutional Review Board of Kampala International University (Ref: KIU-2022-217). Administrative clearance was granted by the Director’s Office of Mbarara Regional Referral Hospital. The study was also registered with the Uganda National Council for Science and Technology (Ref: HS4682ES). Patient consent was waived as the study involved minimal risk and utilized anonymized data.

Quality control

All materials were checked for manufacturer specifications and expiry dates prior to use. Samples were properly labeled to prevent misidentification. Laboratory equipment was sterilized using an autoclave at 121°C for 20 minutes and a hot air oven at 160°C for two hours. Prepared reagents were sterilized at 121°C for 15 minutes and incubated at 37°C for 24 hours to assess sterility. Any contaminated reagents were discarded. Quality control strains *E. coli* ATCC 25922 and *S. aureus* ATCC 25923 were used to verify the performance of antibiotic discs.

## Results

Patient information

Medical charts of 109 patients admitted to the ICU at Mbarara Regional Referral Hospital during the study period were reviewed. The majority of patients were male (68, 62.4%). Most patients were aged 18 to 50 years (46, 42.2%), followed by those younger than 18 years (33, 30.3%). Culture and sensitivity testing was performed in five patients (4.6%).

Overall, 72 patients (66.1%) died during ICU admission. This finding is presented as a descriptive outcome and was not analyzed in relation to antibiotic use or microbiological testing practices (Table 1).

**Table 1:** Demographic and clinical characteristics of ICU patients (N = 109) Demographic characteristics, culture and sensitivity testing status, and clinical outcomes of patients admitted to the ICU. Data are presented as frequencies and percentages. ICU: intensive care unit

Variable	Category	Frequency	Percentage (%)
Age (years)	<18	33	30.3
	18-50	46	42.2
	>50	30	27.5
Gender	Female	41	37.6
	Male	68	62.4
Culture and sensitivity	Yes	5	4.6
	No	104	95.4
Outcome	Died	72	66.1
	Discharged	37	33.9

Antibiotics in the ICU

**Figure 5:**
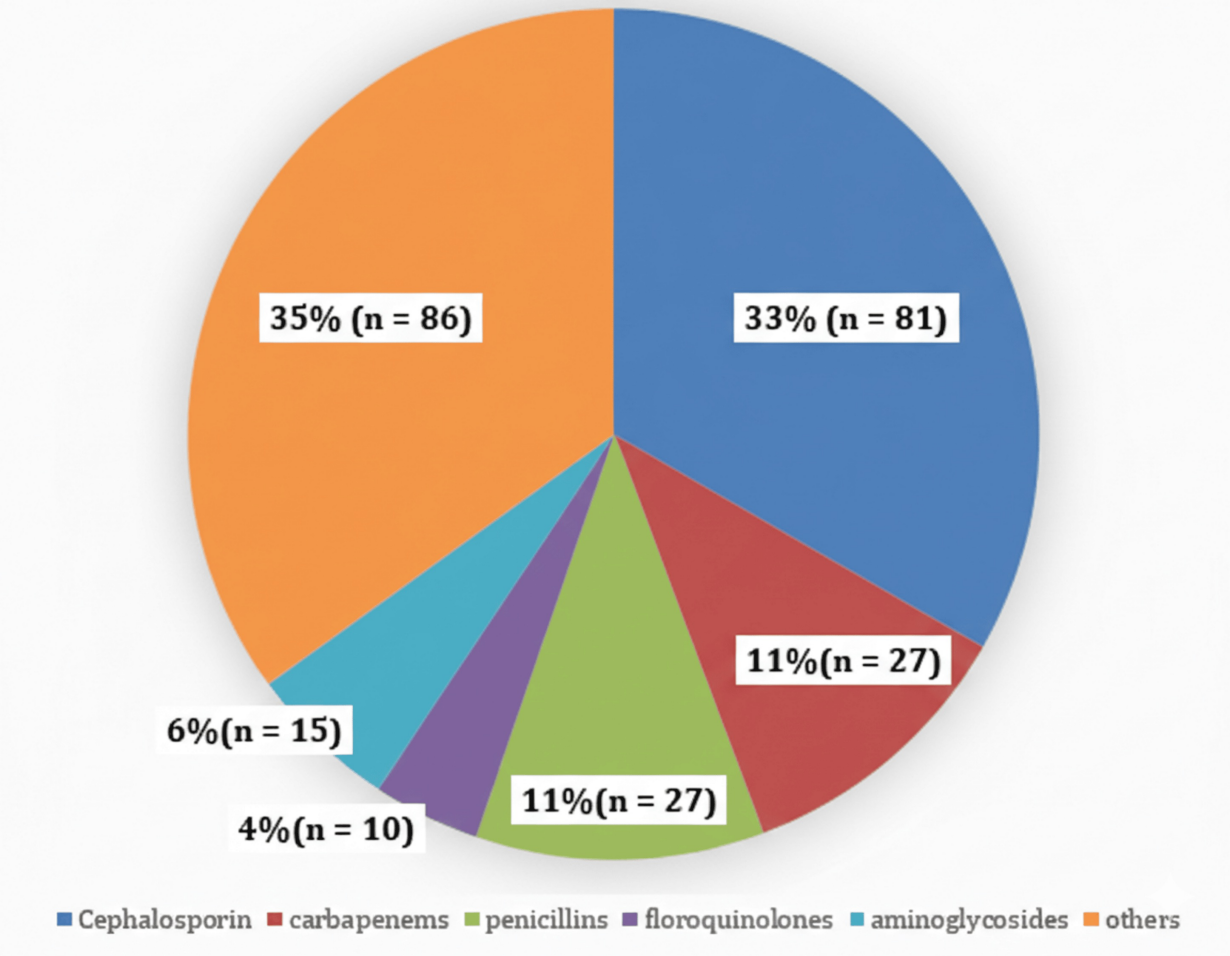
Distribution of antibiotic classes prescribed in the ICU (N = 246) Percentages represent the proportion of each antibiotic class out of a total of 246 antibiotic prescriptions. Absolute frequencies are shown in parentheses. Percentages were rounded to the nearest whole number, and totals may not equal 100% due to rounding. ICU: intensive care unit

All 109 patients received at least one antibiotic, resulting in a total of 246 antibiotic prescriptions comprising 21 different agents. Cephalosporins accounted for the largest proportion of prescriptions (33.0%), mainly ceftriaxone, ceftazidime, and cefixime, followed by carbapenems (11.0%) and penicillins (11.0%) (Figure 5).

Metronidazole was the most frequently prescribed antibiotic (82 prescriptions, 33.3%), followed by ceftriaxone (78, 31.7%). Meropenem and piperacillin-tazobactam were each prescribed 15 times (6.1%), while imipenem was prescribed 12 times (4.9%).

Bacterial isolates

A total of 34 environmental samples were collected from ICU surfaces, of which 32 yielded positive cultures, resulting in 40 bacterial isolates. Gram-positive organisms accounted for 21 isolates (52.5%), predominantly *Bacillus subtilis* (16, 76.2%), followed by *S. aureus* (4, 19.0%). Gram-negative organisms constituted 19 isolates (47.5%), with *Klebsiella* species being the most common (5, 26.3%), followed by *Enterobacter aerogenes* (4, 21.1%) (Table 2).

**Table 2:** Bacterial isolates recovered from ICU environmental surfaces (N = 40) Frequency and percentage distribution of bacterial species isolated from ICU environmental surfaces, including door knobs, taps and sinks, ventilators, beds, oxygen ports, worktops, and trolleys. ICU: intensive care unit

Bacteria species	Door knobs	Tap and sink	Ventilator	Bed	Oxygen port	Worktop	Trolley	N	%
Bacillus subtilis	3	4	2	2	1	1	3	16	40
*Klebsiella *spp.	1	-	-	2	-	1	1	5	12.5
Enterobacter aerogenes	1	2	-	-	-	-	1	4	10
Staphylococcus aureus	1	1	1	1	-	-	-	4	10
Enterobacter cloacae	1	1	1	-	-	-	-	3	7.5
Pseudomonas aeruginosa	-	2	1	-	-	-	-	3	7.5
Escherichia coli	-	1	-	1	-	-	-	2	5
*Proteus *spp.	1	-	1	-	-	-	-	2	5
*Streptococcus *spp.	-	-	-	1	-	-	-	1	2.5
Total	8	11	6	7	1	2	4	40	100

Antimicrobial susceptibility testing was performed on 38 of the 40 isolates. *Klebsiella* species exhibited high resistance to ceftriaxone (5/5), ampicillin (5/5), ceftazidime (4/5), and ciprofloxacin (4/5), with partial sensitivity observed to gentamycin (2/5) and imipenem (2/5). *P. aeruginosa* showed complete resistance to ceftriaxone (3/3) and ampicillin (3/3), but all isolates were sensitive to gentamycin (3/3). *S. aureus* demonstrated high resistance to ceftriaxone, ampicillin, ciprofloxacin, and azithromycin (3/3 each), with partial sensitivity to gentamycin (2/3) and imipenem (2/3).

*E. coli* isolates were resistant to ceftriaxone, ampicillin, and ciprofloxacin (2/2 each), but fully sensitive to gentamycin (2/2). *E. aerogenes* showed complete resistance to ceftriaxone, ampicillin, and ceftazidime (4/4), with partial sensitivity to gentamycin (2/4) and imipenem (2/4) (Table 3).

**Table 3:** Antimicrobial resistance patterns of bacterial isolates Percentage resistance of bacterial isolates to selected antimicrobial agents based on antimicrobial susceptibility testing. Asterisks (*) indicate antibiotics that were not tested against the respective organisms. A: *Klebsiella* spp.; B: *Enterobacter aerogenes*; C: *Enterobacter cloacae*; D: *Pseudomonas aeruginosa*; E: *Staphylococcus aureus*; F: *Streptococcus* spp.; G: *Proteus* spp.; H: *Citrobacter* spp.; I: *Escherichia coli*

Antibiotic class	Antibiotic agents	Organisms (number and % of resistance)								
		A (n = 5)	B (n = 4)	C (n = 2)	D (n = 3)	E (n = 3)	F (n = 1)	G (n = 1)	H (n = 1)	I (n = 2)
Macrolides	Azithromycin	*	*	*	*	100%	100%	*	*	*
Cephalosporins	Ceftazidime	80%	100%	100%	100%	*	*	100%	100%	50%
	Ceftriaxone	100%	100%	100%	100%	100%	100%	100%	100%	100%
Fluoroquinolones	Ciprofloxacin	80%	75%	100%	67%	100%	0%	100%	100%	100%
Penicillins	Ampicillin	100%	100%	100%	100%	100%	100%	100%	100%	100%
	Oxacillin	*	*	*	*	100%	100%	*	*	*
Aminoglycosides	Gentamycin	60%	50%	50%	0%	33.3%	100%	100%	100%	0%
Carbapenems	Imipenem	60%	50%	50%	67%	33%	0%	0%	0%	50%

## Discussion

The baseline cross-sectional survey involved 109 medical files of patients admitted to the ICU from January 2022 to June 2023. The survey aimed at determining the most commonly used antibiotics, the practice of culture and sensitivity to guide antibiotic prescribing, and the clinical outcomes of patients on antibiotic therapy in the ICU. This study did not attempt to establish a relationship between environmental contamination and clinical infections or antibiotic use. The environmental and patient datasets were analyzed independently, and no causal inferences can be drawn. With less than one-third (27.5%) of patients older than 50 years, the current ICU study population was comparable with what was reported by a previous systematic review of Ugandan ICU patients [13]. On the other hand, the current study showed a significantly younger population of ICU admissions compared to several studies elsewhere [14,15]. The probable explanation for this difference is due to a younger population in Uganda with a mean age of 16.3 years, which is much lower compared to most countries. Our study also revealed a much higher proportion of males (62.4%) among the ICU admissions. This is consistent with previous studies, but the reason why men are at higher risk for ICU admission as well as poorer clinical outcomes remains unsettled [16].

The current mortality rate of 66.1% is considerably higher than the 37.5% that was reported in a systematic review of Ugandan adult ICU admissions [13]. This discrepancy might be attributed to the difference between health facilities in their capacities related to healthcare workers and infrastructure to deliver ICU services. The patient overload in tertiary hospitals in low-income compared to high-income countries can contribute to increased mortality in the ICU [15,17]. The high mortality rate in the current study may also reflect resource constraints, including limited access to culture and sensitivity testing, leading to inappropriate antibiotic use.

Our survey showed cephalosporins, carbapenems, and penicillins to be the most commonly prescribed antibiotics in ICU patients. Although all the patients used at least one antibiotic agent, culture and sensitivity test was done only in 5/109 (4.6%) of the patients. The low utilization of culture and sensitivity testing alongside frequent antibiotic prescribing observed in this study highlights potential gaps in antimicrobial stewardship. However, no association between these practices and patient outcomes, including mortality, was assessed in this study. Adequate and timely administration of antibiotics, taking into account local resistance patterns and patient risk factors, is associated with substantially better survival [18].

In this study, 32/34 (94.1%) of the surveyed surfaces in the ICU were contaminated with different species of bacteria. This finding is comparable with 94.7% in Brazil [19] but moderately higher than the 83.3% that was reported in a study in Northern Ethiopia [20].

In this study, *B. subtilis* isolates were the most frequently identified microorganisms in the specimens collected from different surfaces in the ICU, followed by *Klebsiella* isolates, *E. aerogenes*, *S. aureus*, and *P. aeruginosa*. This finding is consistent with a study conducted in an ICU in Bangladesh that showed *Pseudomonas* spp., *E. coli*, *Acinetobacter* spp., and *Klebsiella* spp. [21]. Previous studies also showed *P. aeruginosa* to be a common contaminant on moist surfaces like taps and sinks, leading to it being among the most frequently isolated microorganisms in the ICU [21,22]. *B. subtilis* is one of the most common organisms responsible for environmental contamination in the laboratory [23]. The contamination reported showed that there is a high prevalence of organisms on the tap and sink surfaces, followed by surfaces of door knobs, patients’ beds, and the ventilators. Moist environments may foster biofilm formation, increasing microbial persistence despite cleaning.

Among the predominant bacterial pathogens isolated on surfaces in the ICU, *Klebsiella* isolates and *S. aureus* can survive on surfaces from a few days to a few months [24]. Different bacterial species including *B. subtilis*, *E. coli*, *P. aeruginosa*, *Klebsiella*, and *S. aureus* form biofilms, protecting them from harsh environments and making them difficult to eradicate [24-26]. Moreover, hospitals report insufficient supplies of cleaning agents [27]. Training ICU staff on hygiene protocols and infection prevention procedures may help in mitigating the contamination of surfaces in the ICU and in reducing the transmission of HAIs.

Antimicrobial sensitivity tests of the common bacterial isolates showed a staggeringly high resistance to most of the commonly used antibiotics including ampicillin, oxacillin, ceftriaxone, ceftazidime, and ciprofloxacin but some sensitivity to gentamycin and imipenem. This finding is comparable with what was previously reported by a study in Namibia, where all the isolates from the ICU were resistant to penicillins and cephalosporins [28]. Our finding that *Klebsiella* species showed the highest sensitivity to carbapenems was comparable with previous studies. Frequent and unnecessary use of antibiotics without culture testing may contribute to the observed resistance [29]; however, this was not directly assessed in the present study. Introducing rapid diagnostic kits could enhance timely and appropriate antibiotic selection, potentially reducing resistance rates and improving outcomes.

In parallel, structured antimicrobial stewardship programs supported by systematic quality improvement approaches have been shown to reduce antimicrobial utilization while maintaining clinical outcomes, highlighting the importance of data-driven prescribing practices in hospital settings [30]. Furthermore, preventive strategies such as vaccination may complement antimicrobial stewardship efforts by reducing the incidence of infections and, consequently, the need for antibiotic use, which may help limit the emergence and spread of AMR [30].

Limitations and recommendations for future research

This study has several limitations that should be considered when interpreting the findings. The relatively small number of environmental samples and bacterial isolates limits the generalizability of the results and reduces statistical robustness. In addition, bacterial identification relied on conventional microbiological methods without molecular confirmation, which may have led to misclassification or under-identification of certain organisms. The cross-sectional design, with single-time-point environmental sampling, does not capture temporal variations in microbial contamination.

Furthermore, no analytical linkage was established between environmental isolates and patient-level infections or antibiotic use, limiting inference on transmission dynamics. The retrospective nature of patient data collection may also be affected by incomplete documentation and potential bias in recorded antibiotic use and culture and sensitivity testing practices.

Future research should focus on larger, multicenter studies to enhance generalizability. Incorporation of molecular and genomic techniques would improve the accuracy of organism identification and AMR profiling. Additionally, longitudinal study designs that integrate environmental surveillance with patient-level clinical data are needed to better understand transmission pathways and the relationship between environmental contamination and clinical outcomes.

## Conclusions

Our survey showed younger, male-dominant ICU admissions in our setting. We noted a high rate of antibiotic use but few culture and sensitivity tests in adult ICU admissions, with a considerably higher mortality rate compared to what had been reported in other regions of Uganda and elsewhere. Excessive antibiotic use without confirmation of bacterial infection may increase the risk of emergence and dissemination of AMR in ICU patients. Most of the surveyed surfaces in the ICU were contaminated with different species of bacteria that were mostly isolated from the tap and sink surfaces, followed by surfaces of door knobs, patients’ beds, and ventilators. Most of the bacterial isolates showed a staggeringly high resistance to most of the commonly used antibiotics. We recommend stringent compliance with ICU environmental disinfection and cleaning, strengthening the antimicrobial stewardship program that combines effective infection prevention and control with adequate and timely administration of antibiotics, and taking into account local resistance patterns and individual patient risk factors to improve survival and mitigate the burden of AMR in the ICU. A study with a larger ICU environmental sample size should be done for better generalizability. Establishing a biannual antibiogram for the ICU could guide empirical antibiotic selection and monitor resistance trends.

## References

[1] Antimicrobial Resistance Collaborators (2022). Global burden of bacterial antimicrobial resistance in 2019: a systematic analysis. Lancet.

[2] (2026). WHO. Antimicrobial resistance [Internet]. https://www.who.int/news-room/fact-sheets/detail/antimicrobial-resistance.

[3] Vincent JL, Rello J, Marshall J (2009). International study of the prevalence and outcomes of infection in intensive care units. JAMA.

[4] Vogelaers D, De Bels D, Forêt F, Cran S, Gilbert E, Schoonheydt K, Blot S (2010). Patterns of antimicrobial therapy in severe nosocomial infections: empiric choices, proportion of appropriate therapy, and adaptation rates--a multicentre, observational survey in critically ill patients. Int J Antimicrob Agents.

[5] Sakr Y, Jaschinski U, Wittebole X (2018). Sepsis in intensive care unit patients: worldwide data from the Intensive Care over Nations audit. Open Forum Infect Dis.

[6] WHO WHO (2011). Report on the Burden of Endemic Health Care-Associated Infection Worldwide: Clean Care is Safer Care. https://www.who.int/publications/i/item/report-on-the-burden-of-endemic-health-care-associated-infection-worldwide.

[7] Moolchandani K, Sastry AS, Deepashree R, Sistla S, Harish BN, Mandal J (2017). Antimicrobial resistance surveillance among intensive care units of a tertiary care hospital in Southern India. J Clin Diagn Res.

[8] Ribeiro LF, Lopes EM, Kishi LT (2019). Microbial community profiling in intensive care units expose limitations in current sanitary standards. Front Public Health.

[9] Etemad M, Khani Y, Hashemi-Nazari SS, Izadi N, Eshrati B, Mehrabi Y (2021). Survival rate in patients with ICU-acquired infections and its related factors in Iran's hospitals. BMC Public Health.

[10] Turyatunga G, Gift Wito S, Muwagunzi E (2021). Antibacterial susceptibility patterns of common bacterial species associated with urinary tract infections in patients attending Kam Medical and Diagnostic Centre, Kampala Uganda. SJHR-Africa.

[11] Humphries R, Bobenchik AM, Hindler JA, Schuetz AN (2021). Overview of changes to the Clinical and Laboratory Standards Institute performance standards for antimicrobial susceptibility testing, M100, 31st Edition. J Clin Microbiol.

[12] Kahlmeter G, Brown DF, Goldstein FW (2006). European Committee on Antimicrobial Susceptibility Testing (EUCAST) technical notes on antimicrobial susceptibility testing. Clin Microbiol Infect.

[13] Asiimwe E, Ayoola A, Sabiiti B, Kache S (2022). Adult intensive care in Uganda: a systematic review. [PREPRINT].

[14] Fuchs L, Chronaki CE, Park S (2012). ICU admission characteristics and mortality rates among elderly and very elderly patients. Intensive Care Med.

[15] Wise R, Whittaker R, Garside T (2024). Subtleties and differences of managing ICU patients across South Africa, Australia and UK. Curr Infect Dis Rep.

[16] Lagina M, Ashana DC, Viglianti EM (2024). Sex-based differences in receipt of ICU care: nuances in understanding "less is better?". Crit Care Med.

[17] Li A, Ling L, Qin H (2024). Prognostic evaluation of quick sequential organ failure assessment score in ICU patients with sepsis across different income settings. Crit Care.

[18] Mokrani D, Chommeloux J, Pineton de Chambrun M, Hékimian G, Luyt CE (2023). Antibiotic stewardship in the ICU: time to shift into overdrive. Ann Intensive Care.

[19] Rodrigues DO, da Paixao Peixoto L, Barros ET (2020). Epidemiology of bacterial contamination of inert hospital surfaces and equipment in critical and non-critical care units: a Brazilian study. Microbiol Res J Int.

[20] Darge A, Kahsay AG, Hailekiros H, Niguse S, Abdulkader M (2019). Bacterial contamination and antimicrobial susceptibility patterns of intensive care units medical equipment and inanimate surfaces at Ayder Comprehensive Specialized Hospital, Mekelle, Northern Ethiopia. BMC Res Notes.

[21] Odoyo E, Matano D, Tiria F (2023). Environmental contamination across multiple hospital departments with multidrug-resistant bacteria pose an elevated risk of healthcare-associated infections in Kenyan hospitals. Antimicrob Resist Infect Control.

[22] Salam MT, Bari KF, Gafur DM (2024). Antibiotic resistance pattern in intensive care unit patients of Bangladesh. [PREPRINT].

[23] Konar J, Das S (2013). Common contaminants of bacteriology laboratory: microbiological paramores. Int J Pharm Sci Invent.

[24] López D, Vlamakis H, Kolter R (2010). Biofilms. Cold Spring Harb Perspect Biol.

[25] Otter JA, Vickery K, Walker JT (2015). Surface-attached cells, biofilms and biocide susceptibility: implications for hospital cleaning and disinfection. J Hosp Infect.

[26] Vickery K, Deva A, Jacombs A, Allan J, Valente P, Gosbell IB (2012). Presence of biofilm containing viable multiresistant organisms despite terminal cleaning on clinical surfaces in an intensive care unit. J Hosp Infect.

[27] Alp E, Damani N (2015). Healthcare-associated infections in intensive care units: epidemiology and infection control in low-to-middle income countries. J Infect Dev Ctries.

[28] Shampapi Alphons K, Vanessa Fortune T, Haindongo E, Yapo Guillaume A (2020). Bacterial contamination and antimicrobial susceptibility from the hands of health care workers (HCWs) and inanimate surfaces in the neonatal intensive care unit (NICU) at the Windhoek Central Hospital (WCH). Microbiol Nat.

[29] Bungau S, Tit DM, Behl T, Aleya L, Zaha DC (2021). Aspects of excessive antibiotic consumption and environmental influences correlated with the occurrence of resistance to antimicrobial agents. Curr Opin Environ Sci Heal.

[30] Sallam M, Snygg J (2023). Improving antimicrobial stewardship program using the lean six sigma methodology: a descriptive study from Mediclinic Welcare Hospital in Dubai, the UAE. Healthcare (Basel).

